# Physician clinical management strategies and reasoning: a cross-sectional survey using clinical vignettes of eight common medical admissions

**DOI:** 10.1186/1472-6963-14-176

**Published:** 2014-04-17

**Authors:** Kristofer L Smith, Sarah Ashburn, Jenerius A Aminawung, Micah Mann, Joseph S Ross

**Affiliations:** 1 Department of Medicine, North Shore-Long Island Jewish Health System, New Hyde Park, NY, USA; 2 Hofstra-North Shore School of Medicine, Rego Park, NY, USA; 3 Section of General Internal Medicine, Department of Internal Medicine, Yale University School of Medicine, P.O. Box 208093, 06520 New Haven, CT, USA; 4 Division of Hospital Medicine, The Samuel Bronfman Department of Medicine, Mount Sinai School of Medicine, New York, NY, USA; 5 Section of General Internal Medicine and Robert Wood Johnson Foundation Clinical Scholars Program, Department of Internal Medicine, Yale University School of Medicine and Center for Outcomes Research and Evaluation, Yale-New Haven Hospital, New Haven, CT, USA

## Abstract

**Background:**

Physicians often select clinical management strategies not strongly supported by evidence or guidelines. Our objective was to examine the likelihood of selecting, and rationale for pursuing, clinical management strategies with more or less guideline support among physicians using clinical vignettes of eight common medical admissions.

**Methods:**

We conducted a cross-sectional survey using clinical vignettes of attending physicians and housestaff at one internal medicine program in New York City. Each clinical vignette included a brief clinical scenario and a varying number of clinical management strategies: diagnostic tests, consultations, and treatments, some of which had strong evidence or guideline support (Level 1 strategies) while others had limited evidence or guideline support (Level 3 strategies). Likelihood of selecting a given management strategy was assessed using Likert scales and multiple response options were used to indicate rationale(s) for selections.

**Results:**

Our sample included 79 physicians; 68 (86%) were younger than 40 years of age, 34 (43%) were female. There were 31 attending physicians (39%) and 48 housestaff (61%) and 39 (49%) had or planned to have primarily primary care internal medicine clinical responsibilities. Overall, physicians were more likely to select Level 1 strategies “always” or “most of the time” when compared with Level 3 strategies (82% vs. 43%; p < 0.001), with wide variation across the eight medical admissions. There were no differences between attending and housestaff physician likelihood of selecting Level 3 strategies (47% vs. 45%, p = 0.36). Supportive evidence and local practice patterns were the two most common rationales behind selections; supportive evidence was cited as the most common rationale for selecting Level 1 when compared with Level 3 strategies (63% versus 30%; p < 0.001), whereas ruling out other severe conditions was cited most often for Level 3 strategies.

**Conclusions:**

For eight common medical admissions, physicians selected more than 80% of management strategies with strong evidence or guideline support, but also selected more than 40% of strategies for which there was limited evidence or guideline support. The promotion of evidence-based care, including the avoidance of care that is not strongly supported by evidence or guidelines, may require better evidence dissemination and educational outreach to physicians.

## Background

The American health care system suffers from uneven quality [1] and wide variation in utilization [2]; both of which contribute to an impending crisis of increased health care spending [3]. Clinical practice guidelines are formulated in effort to improve the quality of care and to decrease inappropriate care and wasteful healthcare cost [4-6]. However, more than two decades following the introduction of the first guideline, many physicians and other clinicians fail to abide by these professional recommendations [7,8]. Partly this may be a consequence of varying satisfaction with guidelines among physicians [9,10], although explanations for poor physician adherence to guideline-recommended care has also included lack of awareness [11], difficulty changing entrenched practice habits [12], disagreement with guideline recommendations [13], system or environmental constraints [14], and shortcomings of using guidelines to inform the care of patients with multiple comorbidities [15,16].

While there is a rich literature on general guideline attitudes and adherence, the differences in likelihood and reasoning for selecting between clinical management strategies that have different levels of guideline support has been sparingly investigated. Within most guidelines there is a gradation of recommendations from those with high quality evidence to those with little or no evidence of efficacy. Moreover, these selections may be different for management strategies that are focused on treatments, as opposed to imaging, laboratory testing, or consultations.

Understanding whether physicians use different justifications or explanations for selecting evidence-based, highly supported medical care versus poorly supported, or even contraindicated, care could have substantial implications for reducing the provision of wasteful and potentially dangerous medical care. To investigate differences in likelihood of selecting, and rationale for pursuing, clinical management strategies with more or less guideline support among physicians, we conducted a cross-sectional study using clinical vignettes of common medical admissions among attending and housestaff physicians at one academic medical center.

## Methods

### Design and setting

We developed a cross-sectional, self-administered survey for physicians and housestaff working within the Department of Internal Medicine at the Mount Sinai Hospital, a large tertiary care academic medical center in New York City, New York, USA. The Department has 400 full-time faculty, 140 categorical, preliminary, and research track housestaff, and more than 10,000 annual hospital discharges. At the time the study was conducted, all investigators (with the exception of JA) were either on the faculty or trainees at the institution.

### Study subjects

Attending physicians in the Department of Internal Medicine who worked at least 4-weeks per year on the general medicine or geriatrics teaching services and housestaff in the Department of Internal Medicine at the post-graduate year two level and above were eligible. Participation was voluntary and participants received a $5 appreciation gift card. This study was approved by the Mount Sinai School of Medicine Institutional Review Board and all study subjects underwent informed consent.

### Clinical vignette development and other survey information

We designed a survey to examine physician clinical decision making for eight common medical admissions. All chosen medical admissions were conditions for which published evidence-based guidelines were available that rated the strength of existing evidence supporting the clinical recommendations. The eight chosen conditions were congestive heart failure [17,18], chronic obstructive pulmonary disease [19], transient ischemic attack [20-22], community acquired pneumonia [23], asthma [24], acute coronary syndrome [25], syncope [26,27], and end-stage lung cancer [28,29]. Clinical vignettes were developed that provided a straightforward presentation of the medical condition. The intent was for the diagnosis to be clear to both attending physicians and housestaff (see Additional file 1 for complete vignettes).

Each vignette was constructed through an iterative process by three of the authors (KS, MM, JSR), which included solicited feedback from a second general internist and an appropriate subspecialist as to whether the clinical scenario and decisions in the vignettes were ambiguous. We then piloted the survey on three housestaff and two attending physicians and feedback was incorporated into the final vignettes. Our eight vignettes each had two parts. Part one described the initial patient presentation to the hospital and a series of possible clinical management strategies from all four core areas of patient care, including treatments, imaging, laboratory tests, and consultations. Part two presented results of the diagnostic tests included in part one and additional clinical management strategies. All responses for part one were collected before participants received the second part of the vignettes, so part one responses could not be changed after receiving additional information during part two.

The likelihood of selecting a clinical management strategy was asked using a 5-point ordinal scale (always/almost always, most of the time, some of the time, rarely, never). For each selection, respondents were asked to indicate one or more rationales, including: local practice patterns, supervisor expectations, supporting data or guidelines (evidence), malpractice concerns, academic purposes (i.e., as part of a learning exercise), or to rule out more serious conditions. These rationales were derived from several group sessions with attending and housestaff physicians where clinical management strategies were discussed. Each vignette listed clinical strategies for which there was a range of evidence. We included management strategies that were either recommended or not recommended by the guidelines, based on clinical evidence or expert opinion. Recommended strategies included both class I and II recommendations (including evidence levels A and B and C and D, respectively) and are henceforth referred to as Level 1 strategies; non-recommended strategies include class III recommendations (including evidence levels A, B, C and D) and are henceforth referred to as Level 3 strategies. In particular, class I and II guidelines recommend management strategies for which the benefits are greater than or equal to the risk, whereas class III guidelines suggest that the risk outweighs the benefits. In all, the 8 clinical vignettes included 64 Level 1 strategies and 78 Level 3 strategies.

We also collected information about demographic and professional characteristics of respondents such as: age, gender, intended or current subspecialty, and intended or current clinical activities. Respondents were also asked to rate factors that influence their practice patterns using a 5-point ordinal scale and sources of information that inform their clinical practice on a 7-point ordinal scale. Housestaff and attending physicians completed the surveys during regularly scheduled noon-conferences and administrative time respectively.

### Statistical analysis

Responses to the likelihood of making a Level 1 or Level 3 clinical strategies were collapsed into two groups; “Always” and “Most of the time” versus “Some of the time”, “Rarely” and “Never.” The proportion of respondents selecting Level 1 and Level 3 strategies ‘always’ and ‘most of the time’ was calculated for each clinical scenario. Physicians’ decision selection within each of the four core areas of care; laboratory testing, imaging, treatment and consult decisions, and the rationales behind selected clinical decisions were determined. We compared the proportions of respondents that selected “All” or “Most” of the time for Level 1 and Level 3 decisions by demographic and clinical practice characteristics using Chi Squared-test and Mann–Whitney U-test as appropriate. As the clinical scenarios varied in disease and severity of presentation, to inform future work, we also compared how often Level 1 and Level 3 management strategies were selected within each vignette and in comparison across clinical scenarios, using community acquired pneumonia as the reference scenario. Finally, the rationales for were compared for selecting Level 1 and Level 3 clinical management strategies. We used a type 1 error level of 0.01 to account for multiple comparisons. All analyses were completed using SPSS® version 18.0 (PASW Statistics, Chicago, IL).

## Results

Of the 129 physicians eligible for the study, 79 (61%) completed the survey; made up of 48 (61%) housestaff and 31 (39%) attending physicians. Sixty eight (86%) physicians were younger than 40 years of age and 34 (43%) were female (Table 1). Gender distribution was similar among attending physicians and housestaff that responded to the survey. Sixty eight (94%) of the physicians identified their current or former training as categorical or primary care internal medicine and half (51%) of them had or intended to have primary care clinical responsibilities.

**Table 1:** Respondent physician characteristics (N = 79)

**Physician characteristics**	N (%)*
**Age**	
< 40 years	68 (86)
40 to 60 years	11 (14)
**Gender**	
Male	45 (57)
Female	34 (43)
**Physician level of practice**	
Housestaff	48 (61)
Attending	31 (39)
**Current or planned clinical practice**	
Primary care internal medicine	40 (51)
Internal medicine subspecialty	37 (47)
Undecided	2 (3)
**Current or planned practice location**	
Metropolitan	74 (94)
Suburban	7 (9)
Rural	0 (0)
**Self-Reported factors influencing clinical practice**^**†**^	
Supportive evidence	76 (96)
Prior or local practice patterns (culture)	71 (90)
Prior experience	56 (71)
Patient demand	42 (53)
Malpractice concern	29 (37)
Hospital profit	14 (18)
Individual profit	9 (11)
**Sources of information used to inform clinical practice**^**†**^	
Peer-reviewed journals	64 (81)
Practice guidelines	60 (76)
Prior experience	44 (56)
Other resources	36 (46)
Continuing medical education events (local and national)	9 (11)

*Percentages may not sum to 100 because of rounding.

^†^Responses not mutually exclusive.

Almost all (94%) of the respondents indicated their preferred practice setting as metropolitan, with none of the respondents having any intentions of practicing in a rural setting. When asked to describe factors that influence their clinical practice, the most common reasons cited were accumulated research evidence (n = 76; 96%), hospital culture (n = 71; 90%) and individual practice experience (n = 56; 71%). Only a third cited malpractice concerns (n = 29; 37%). When asked about the main sources of information used to inform clinical practice, the most common sources cited were peer-reviewed journals (n = 64; 81%) and practice guidelines (n = 60; 76%).

### Clinical management strategies

Overall, physicians were more likely to select Level 1 management strategies “always” or “most of the time” when compared with Level 3 management strategies (82% vs. 43%; p < 0.001) (Table 2). However, physicians were less likely to select Level 1 strategies for vignettes focused on care for end-stage lung cancer, transient ischemic attack, and chronic obstructive pulmonary disease when compared with the vignette for community acquired pneumonia (Table 2). Similarly, physicians were more likely to select Level 3 strategies for vignettes focused on care for acute coronary syndrome, asthma, end-stage lung cancer, transient ischemic attack, and chronic obstructive pulmonary disease when compared with the vignette for community acquired pneumonia (Table 2). Housestaff and attending physicians were equally likely to choose Level 1 strategies (83% vs. 81%, p = 0.22) and Level 3 strategies (47% vs. 45%, p = 0.36). There was no difference in selection of Level 1 or Level 3 strategies by physicians’ gender, age, training, or intended clinical responsibilities (p values > 0.10; data not shown).

**Table 2:** Proportion of respondents selecting Level 1 and Level 3 clinical management strategies ‘always’ or ‘most of the time’, overall and stratified by each of the 8 clinical vignettes (N = 79)

	**Level 1 strategies, %**	**Level 3 strategies, %**	**P value**
**Overall**	82	43	< 0.001
**Clinical vignette scenario**			
Community acquired pneumonia	93	25	< 0.001
Acute coronary syndrome*	93	65	< 0.001
Congestive heart failure	85	31	< 0.001
Syncope	83	33	< 0.001
Asthma*	81	52	< 0.001
End-Stage lung cancer*^†^	77	61	0.01
Transient ischemic attack*^†^	73	17	< 0.001
Chronic obstructive pulmonary disease*^†^	69	57	0.08

*Proportion of respondents that selected Level 3 clinical management strategies was significantly different that the proportion that selected Level 3 strategies for the community acquired pneumonia vignette.

^†^Proportion of respondents that selected Level 1 clinical management strategies was significantly different that the proportion that selected Level 1 strategies for the community acquired pneumonia vignette.

### Rationale for selection clinical management strategies

**Figure 1:**
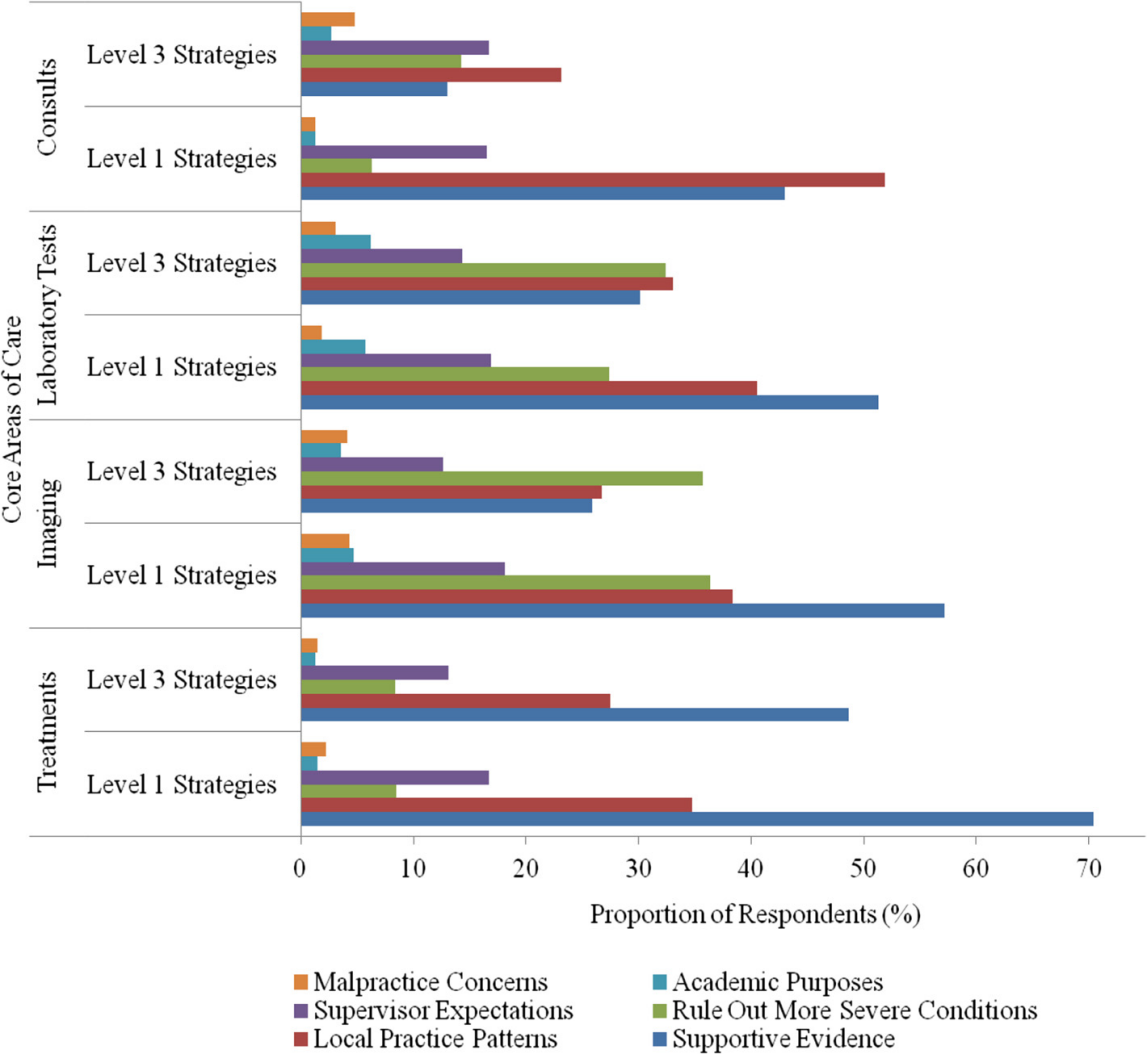
Proportion of respondents citing selected rationale for selecting Level 1 and Level 3 clinical management strategies, stratified by core areas of care: treatments, imaging, laboratory tests, and consults (N = 79).

The two most common rationales for clinical management strategies within the four core areas of care were supportive evidence and local practice patterns (Figure 1). Evidence was cited as the most common rationale for selecting Level 1 strategies when compared with Level 3 strategies (63% versus 30%; p < 0.001). Although ruling out other severe conditions was cited more often for Level 3 strategies, there was no significant difference in the selection of either Level 1 or 3 clinical strategies for the other queried rationales (Table 3). Rationales cited by physicians for their clinical management strategies were similar by physicians’ gender and training (data not shown). Physicians who had or planned to have clinical practices that included primary care were less likely to cite supervisor expectations as the rationale for selecting either Level 1 strategies (9% vs. 27%; p < 0.001) or Level 3 strategies (7% vs. 23%; p < 0.001) (Table 4), as were physicians whose clinical practice was self-reported to be influenced by malpractice concerns (1% vs. 21% and 1% vs. 17% for Level 1 and Level 3 decisions, respectively; p values ≤ 0.005).

**Table 3:** Proportion of respondents citing selected rationale for Level 1 and Level 3 clinical management strategies (N = 79)

**Selected rationale**	**Overall, %**	**Level 1 strategies, %**	**Level 3 strategies, %**	**P value**
Supportive evidence	45	63	30	< 0.001
Local practice patterns	32	37	29	0.23
Rule out more serious conditions	22	18	26	0.17
Supervisor expectations	15	17	14	0.56
Academic purposes	4	3	4	0.70
Malpractice concerns	3	2	3	0.65

**Table 4:** Rationale for selecting Level 1 or Level 3 clinical management strategies, stratified by physician characteristics and self-reported factors that influence clinical practice (N = 79)

**Selected rationale**	**Clinical strategy**	**Physician characteristics**						**Self-reported factors that influence clinical practice**					
		**Clinical practice includes primary care**			**Attending physician**			**Malpractice concerns**			**Practice guidelines**		
		**Yes, %**	**No, %**	**P value**	**Yes, %**	**No, %**	**P value**	**Yes, %**	**No, %**	**P value**	**Yes, %**	**No, %**	**P value**
**Supportive evidence**	**Level 1**	64	62	0.97	64	62	0.67	57	64	0.32	63	64	0.87
	**Level 3**	30	30	0.70	31	29	0.72	29	30	0.79	30	31	0.81
**Local practice patterns**	**Level 1**	38	38	0.80	31	41	0.16	12	43	< 0.001	37	38	0.98
	**Level 3**	29	28	0.94	27	30	0.39	11	32	< 0.001	28	31	0.43
**Rule out more serious conditions**	**Level 1**	19	17	0.17	20	17	0.15	27	16	0.18	19	14	0.17
	**Level 3**	27	25	0.51	28	25	0.26	31	25	0.53	28	20	0.13
**Supervisor expectations**	**Level 1**	9	26	< 0.001	3	26	< 0.001	1	21	0.003	21	4	0.004
	**Level 3**	6	22	< 0.001	2	21	< 0.001	1	17	0.005	17	5	0.02
**Academic purposes**	**Level 1**	2	3	0.26	1	3	0.40	1	3	0.36	3	1	0.16
	**Level 3**	3	3	0.69	2	4	0.20	2	4	0.33	4	2	0.43
**Malpractice concerns**	**Level 1**	2	4	0.02	1	4	0.02	1	4	0.11	4	1	0.04
	**Level 3**	3	5	0.10	2	5	0.01	2	4	0.13	5	2	0.36

## Discussion

In our cross sectional study of physician clinical management strategies for eight common medical admissions at a single hospital, physicians chose strategies with strong evidence or guideline support 82% of the time, but also selected strategies for which there was limited evidence more than 40% of the time, with wide variation across the eight medical conditions. In our study these rates did not vary between attending and housestaff physicians. Although these attending and housestaff physicians trained at a variety of institutions, their similar self-reported practice styles may speak to the impact of local practice culture on clinical reasoning, particularly when selecting clinical strategies without evidence or that are unsupported by expert guidelines.

When asked to indicate a reason for selecting evidence-supported clinical management strategies, almost 75% of physicians cited that evidence supported their selection. On the other hand, when asked to indicate a reason for selecting non-evidence-supported clinical management strategies, supportive evidence was cited less frequently and local practice culture was indicated as a rationale more often.

These discretionary clinical management selections may have substantial cost, including potential for patient harm, and likely do not provide patients much benefit. One possibility is that physicians either misunderstand the evidence or are simply following the lead of the community within which they practice. Better dissemination of evidence and educational outreach to physicians, as well as attention to local practice culture, will be essential to the promotion of evidence-based care, including the avoidance of care that is not strongly supported by evidence or guidelines. As medicine grapples with the imperative of reigning in excess utilization of care and the associated costs, the Choosing Wisely campaign has helped medical professional societies identify commonly used medical tests and procedures that are not supported by clinical evidence and may be unnecessary, and in some instances can cause harm. The goal of the campaign is to encourage physicians, patients and other healthcare stakeholders to think and talk about how to make wise decisions about the most appropriate care based on a patients’ individual situation [30]. As this campaign continues to grow, our study demonstrates the challenge of influencing clinical decision making, as selection of non-evidence-based, Level 3 strategies was common in our clinical vignettes and is often justified for reasons beyond supportive evidence, such as ruling out other severe conditions.

Interestingly, neither housestaff nor attending physicians felt that their decisions were driven by medical malpractice concerns. Even when physicians chose an unsupported option, less than 5% of the time did they indicate that their thinking was driven by legal considerations. These data suggest that while medical malpractice has become a popular target for new cost containment policy, physician behavior may be driven more strongly by other factors. If this is true, the impact of tort reform on utilization may be more modest than anticipated.

While this study had a high response rate and gathered data across a wide-selection of common conditions, there are important limitations to consider. Our study employed a survey based on hypothetical clinical patients as a means to investigate clinical reasoning. While the cases were designed to be unambiguous, straight-forward, and based on available guidelines, respondents may not have found them to be a realistic proxy for clinical practice or may simply have exhibited a Hawthorne effect and would act differently in real life. In this case, our study likely represents conservative estimates of practice and our finding that more than 80% of Level 1 strategies were selected, and more than 40% of Level 3 strategies were selected, is a best case scenario. Second, the survey was not a validated tool to measure clinical management.

However, the survey was developed by several clinicians with expertise in clinical management and decision making and was piloted to test ease of interpretation and feasibility. Third, many patients admitted to a hospital are often more complex than the presented clinical vignettes, with multiple possible diagnoses, making identifying the clearly correct strategy and subsequent treatment plan difficult. Future work could attempt to identify actual strategies selected for patients with these common medical conditions and then survey the reason for provider selections. Finally, because guidelines are not uniform, the highest ranked clinical strategies for one clinical condition may not be as strongly supported as the highest ranked clinical strategies for another. This may explain the wide variation in decision making across clinical conditions that we observed. As guidelines become more uniform and become less embedded in specialty societies, the different categories of evidence will hopefully be more easily compared across guidelines.

## Conclusions

For eight common medical admissions, physicians selected more than 80% of clinical management strategies for which there was strong evidence or guideline support. However, they also selected more than 40% of management options for which there was limited evidence or guideline support. As evidence was the most common reason for selecting guideline supported strategies, the promotion of evidence-based care, including the avoidance of care that is not strongly supported by evidence or guidelines, may require better evidence dissemination and educational outreach to physicians.

## Competing interests

JSR receives research support from Medtronic, Inc. and Johnson and Johnson, Inc. to develop methods to promote data sharing and from the U.S. Centers of Medicare and Medicaid Services (CMS) to develop and maintain hospital performance measures that are used for public reporting; he is also a member of a scientific advisory board for FAIR Health, Inc. No other authors have potential competing interests to report.

## Authors’ contributions

KLS and JSR were responsible for the conception and design of this work and drafted the manuscript. KLS, SA, and MM were responsible for acquisition of data. JA conducted the statistical analysis. JSR provided supervision. KLS, SA, JA, MM, and JSR participated in the analysis and interpretation of the data and critically revised the manuscript for important intellectual content. All authors read and approved the final manuscript.

## Pre-publication history

The pre-publication history for this paper can be accessed here:

http://www.biomedcentral.com/1472-6963/14/176/prepub

## Supplementary Material

Additional file 1 **Triple C manuscript 2013-10-16 appendix.doc, 502 K **http://www.biomedcentral.com/imedia/1827684101160557/supp1.doc. Click here for file

## Related paper presentation

Poster Presentation at the Society General Internal Medicine Annual Meeting. Phoenix, AZ May 2011.

## References

[B1] Yasaitis L Fisher ES Skinner JS Chandra A Hospital quality and intensity of spending: is there an association? Health Aff (Millwood) 2009 14 4 w566 w572 10.1377/hlthaff.28.4.w566 19460774 PMC2768577

[B2] Medicare Payment Advisory Commission (U.S.) Measuring Regional Variation in Service Use Report to the Congress 2009 Washington: U.S. G.P.O 24

[B3] Chernew ME Hirth RA Cutler DM Increased spending on health care: long-term implications for the nation Health Aff (Millwood) 2009 14 5 1253 1255 10.1377/hlthaff.28.5.1253 19738238

[B4] Field MJ Lohr KN Institute of Medicine (U.S.). Committee to Advise the Public Health Service on Clinical Practice Guidelines., United States. Dept. of Health and Human Services Clinical practice guidelines: directions for a new program 1990 Washington, D.C.: National Academy Press

[B5] Chassin MR Practice guidelines: best hope for quality improvement in the 1990s J Occup Med 1990 14 12 1199 1206 10.1097/00043764-199012000-00015 2292739

[B6] McCabe C Kirchner C Zhang H Daley J Fisman D Guideline-concordant therapy and reduced mortality and length of stay in adults with community-acquired pneumonia: playing by the rules Arch Intern Med 2009 14 16 1525 1531 10.1001/archinternmed.2009.259 19752411

[B7] Pires LA Ganji JR Jarandila R Steele R Diagnostic patterns and temporal trends in the evaluation of adult patients hospitalized with syncope Arch Intern Med 2001 14 15 1889 1895 10.1001/archinte.161.15.1889 11493131

[B8] Arnold FW LaJoie AS Brock GN Peyrani P Rello J Menendez R Lopardo G Torres A Rossi P Ramirez JA Improving outcomes in elderly patients with community-acquired pneumonia by adhering to national guidelines: community-acquired pneumonia organization international cohort study results Arch Intern Med 2009 14 16 1515 1524 10.1001/archinternmed.2009.265 19752410

[B9] Farquhar CM Kofa EW Slutsky JR Clinicians’ attitudes to clinical practice guidelines: a systematic review Med J Aust 2002 14 9 502 506 12405894 10.5694/j.1326-5377.2002.tb04920.x

[B10] Formoso GLAMN Practice guidelines: useful and “participative” method?: survey of italian physicians by professional setting Arch Intern Med 2001 14 16 2037 2042 10.1001/archinte.161.16.2037 11525707

[B11] Wigder HN Arai DA Narasimhan K Cohan S ACEP chest pain policy: emergency physician awareness Ann Emerg Med 1996 14 5 606 609 10.1016/S0196-0644(96)70164-6 8629782

[B12] Main DS Cohen SJ DiClemente CC Measuring physician readiness to change cancer screening: preliminary results Am J Prev Med 1995 14 1 54 58 7748587

[B13] Tunis SR Hayward RS Wilson MC Rubin HR Bass EB Johnston M Steinberg EP Internists’ attitudes about clinical practice guidelines Ann Intern Med 1994 14 11 956 963 10.7326/0003-4819-120-11-199406010-00008 8172440

[B14] Weinberger M Saunders AF Samsa GP Bearon LB Gold DT Brown JT Booher P Loehrer PJ Breast cancer screening in older women: practices and barriers reported by primary care physicians J Am Geriatr Soc 1991 14 1 22 29 1987253 10.1111/j.1532-5415.1991.tb05901.x

[B15] Boyd CM Darer J Boult C Fried LP Boult L Wu AW Clinical practice guidelines and quality of care for older patients with multiple comorbid diseases: implications for pay for performance JAMA 2005 14 6 716 724 10.1001/jama.294.6.716 16091574

[B16] Cabana MD Rand CS Powe NR Wu AW Wilson MH Abboud PA Rubin HR Why don’t physicians follow clinical practice guidelines? a framework for improvement JAMA 1999 14 15 1458 1465 10.1001/jama.282.15.1458 10535437

[B17] Jessup M Abraham WT Casey DE Feldman AM Francis GS Ganiats TG Konstam MA Mancini DM Rahko PS Silver MA Stevenson LW Yancy CW 2009 focused update: ACCF/AHA guidelines for the diagnosis and management of heart failure in adults: a report of the American college of cardiology foundation/american heart association task force on practice guidelines: developed in collaboration with the international society for heart and lung transplantation Circulation 2009 14 14 1977 2016 19324967 10.1161/CIRCULATIONAHA.109.192064

[B18] Hunt SA Abraham WT Chin MH Feldman AM Francis GS Ganiats TG Jessup M Konstam MA Mancini DM Michl K Oates JA Rahko PS Silver MA Stevenson LW Yancy CW 2009 focused update incorporated into the Acc/Aha 2005 guidelines for the diagnosis and management of heart failure in adults: a report of the American college of cardiology foundation/american heart association task force on practice guidelines developed in collaboration with the international society for heart and lung transplantation J Am Coll Cardiol 2009 14 15 e1 e90 10.1016/j.jacc.2008.11.013 19358937

[B19] Rabe KF Hurd S Anzueto A Barnes PJ Buist SA Calverley P Fukuchi Y Jenkins C Rodriguez-Roisin R van Weel C Zielinski J Global strategy for the diagnosis, management, and prevention of chronic obstructive pulmonary disease Am J Respir Crit Care Med 2007 14 6 532 555 10.1164/rccm.200703-456SO 17507545

[B20] Johnston SC Nguyen-Huynh MN Schwarz ME Fuller K Williams CE Josephson SA Hankey GJ Hart RG Levine SR Biller J Brown RD Jr Sacco RL Kappelle LJ Koudstaal PJ Bogousslavsky J Caplan LR van Gijn J Algra A Rothwell PM Adams HP Albers GW National stroke association guidelines for the management of transient ischemic attacks Ann Neurol 2006 14 3 301 313 10.1002/ana.20942 16912978

[B21] Easton JD Saver JL Albers GW Alberts MJ Chaturvedi S Feldmann E Hatsukami TS Higashida RT Johnston SC Kidwell CS Lutsep HL Miller E Sacco RL Definition and evaluation of transient ischemic attack Stroke 2009 14 6 2276 2293 10.1161/STROKEAHA.108.192218 19423857

[B22] Adams RJ Albers G Alberts MJ Benavente O Furie K Goldstein LB Gorelick P Halperin J Harbaugh R Johnston SC Katzan I Kelly-Hayes M Kenton EJ Marks M Sacco RL Schwamm LH Update to the AHA/ASA recommendations for the prevention of stroke in patients with stroke and transient ischemic attack Stroke 2008 14 5 1647 1652 10.1161/STROKEAHA.107.189063 18322260 PMC4198335

[B23] Mandell LA Wunderink RG Anzueto A Bartlett JG Campbell GD Dean NC Dowell SF File TM Jr Musher DM Niederman MS Torres A Whitney CG Infectious diseases society of America/American thoracic society consensus guidelines on the management of community-acquired pneumonia in adults Clin Infect Dis 2007 14 Suppl 2 S27 S72 17278083 10.1086/511159 PMC7107997

[B24] National Heart L, and Blood Institute, U.S. National Institutes of Health. National Asthma Education and Prevention Program Guidelines for the Diagnosis and Management of Asthma 2007 Available at: http://www.nhlbi.nih.gov/guidelines/asthma/. Accessed May 31, 2012

[B25] Anderson JL Adams CD Antman EM Bridges CR Califf RM Casey DE Chavey WE Fesmire FM Hochman JS Levin TN Lincoff AM Peterson ED Theroux P Wenger NK Wright RS ACC/AHA 2007 guidelines for the management of patients with unstable angina/non ST-elevation myocardial infarction: a report of the American College of Cardiology/American Heart Association Task Force on Practice Guidelines (Writing Committee to Revise the 2002 Guidelines for the Management of Patients With Unstable Angina/Non ST-Elevation Myocardial Infarction): developed in collaboration with the American College of Emergency Physicians, the Society for Cardiovascular Angiography and Interventions, and the Society of Thoracic Surgeons: endorsed by the American Association of Cardiovascular and Pulmonary Rehabilitation and the Society for Academic Emergency Medicine Circulation 2007 14 7 803 877 10.1161/CIRCULATIONAHA.107.185752 17679616

[B26] Brignole M Alboni P Benditt DG Bergfeldt L Blanc J-J Thomsen PEB van Dijk JG Fitzpatrick A Hohnloser S Janousek J Kapoor W Kenny RA Kulakowski P Masotti G Moya A Raviele A Sutton R Theodorakis G Ungar A Wieling W Guidelines on management (diagnosis and treatment) of syncope â€ Eur Heart J 2004 14 22 2054 2072 11465961 10.1053/euhj.2001.2739

[B27] Moya ASR Ammirati F Blanc JJ Brignole M Dahm JB Deharo JC Gajek J Gjesdal K Krahn A Massin M Pepi M Pezawas T Ruiz Granell R Sarasin F Ungar A van Dijk JG Walma EP Wieling W task force for the diagnosis and management of syncope; european society of cardiology (ESC); european heart rhythm association (EHRA); heart failure association (HFA); heart rhythm society (HRS),Guidelines for the diagnosis and management of syncope (version 2009) Heart J 2009 14 21 2631 2671 10.1093/eurheartj/ehp298 PMC3295536 19713422

[B28] Griffin JP Koch KA Nelson JE Cooley ME Palliative care consultation, quality-of- life measurements, and bereavement for end-of-life care in patients with lung cancer Chest 2007 14 3 suppl 404S 422S 17873182 10.1378/chest.07-1392

[B29] Kvale PA Selecky PA Prakash UBS Palliative care in lung cancer Chest 2007 14 3 suppl 368S 403S 17873181 10.1378/chest.07-1391

[B30] American Board of Internal Medicine Foundation Choosing Wisely Available at: http://www.choosingwisely.org/. Accessed October 7, 2013

