# Supplementary material for: Physician clinical management strategies and reasoning: a cross-sectional survey using clinical vignettes of eight common medical admissions
Source: BMC Health Serv Res. 2014 Apr 17;14:176. doi: 10.1186/1472-6963-14-176 (PMC4021187; doi:10.1186/1472-6963-14-176)
Supplement: Additional file 1 — Triple C manuscript 2013-10-16 appendix.doc, 502 K http://www.biomedcentral.com/imedia/1827684101160557/supp1.doc. [file 1472-6963-14-176-S1.doc]

**Creating Competent Clinicians (TRIPLE C)**

An Accreditation Council for Graduate Medical Education (ACGME) requirement for all residency programs in Internal Medicine is that the competencies of practice based learning and improvement must be integrated into the training experience. A means for satisfying this requirement and improving our training program is to survey both residents and their teaching attending, using hypothetical clinical vignettes to assess adherence to practice based guidelines.

The clinical vignettes have two parts. The first part describes an acute presentation of a patient illness, along with any relevant history of illness. After reviewing the details of the patient presentation you will be asked to indicate your preferences for further evaluation, ordered alphabetically. You will also be asked to indicate the reasons for your decisions.

The second part of the vignette will reveal the results of appropriate diagnostic evaluations (regardless of your choices as per what you would order). You will then be asked to indicate your preferences for further tests and treatments. You will again be asked to indicate the reasons for your decisions.

**For all decisions, please check all the reasons that apply.**

**Please base your responses on your real-life experience of clinical care as delivered at Mount Sinai Hospital.**

Participation in this survey is entirely voluntary.  The data will be collected anonymously and will only be shared in aggregate.  The benefits to participation in this survey are that the residency program will achieve a better understanding of house staff clinical strengths and weaknesses and aggregate data will allow the program, in a more targeted fashion, to address any weaknesses.  This survey will take 20-30 minutes to complete. You will be given that time during this administrative session/morning report.  Thank you for your consideration.

Sincerely,

Mark Babyatsky,

Kristofer Smith

Micah Mann

Joseph Ross

GCO# # 09-1076

Exempted from IRB Review Category 2

**----------Page Intentionally Left Blank----------**

**Section I: Part 1 of Clinical Vignettes A-H**

In this section, you will be given an acute presentation of patient illness, along with any relevant history of illness for 8 commonly encountered hospital conditions. After reviewing the details of the patient presentation you will be asked to indicate the likelihood of arranging for series of further evaluations, ordered alphabetically. **Please check all that apply.** You will also be asked to indicate the reason for your decisions. Once you have completed part 1 of all 8 cases, you will be asked to return this booklet and you will be given a second booklet with part 2 of the clinical vignettes.

Clinical Vignette A, Part 1

A 75 year old male with a past medical history of NYH Class III congestive heart (Echo from admission 6 months prior shows RV and LV dilation and an LVEF of 28%) on optimal medical management, status post AICD placement, chronic kidney disease stage III, coronary artery disease status post a non-drug eluting stent to the mid-LAD 2 years prior, and hypertension is admitted through the emergency room with 7 days of shortness of breath, decreased exercise tolerance and increased lower extremity edema. The patient denies any chest pain. Vital signs reveal a temperature of 37.2, blood pressure of 135/84, heart rate of 92, respiratory rate of 20, oxygen saturation 93% on room air. Physical exam is significant for mild respiratory distress, bilateral crackles at lung bases, +JVD, and warm extremities with 1+ pitting lower extremity edema bilaterally. This patient has had 3 admissions for congestive heart failure in the past 6 months.

Based on the findings above, how often would you order the following:

|  | Always/ almost always | Most of the time | Some of the time | Rarely | Never |
| --- | --- | --- | --- | --- | --- |
| 1. **Arterial blood gas** |  |  |  |  |  |
| 1. **Basic metabolic panel (chem 7)** |  |  |  |  |  |
| 1. **BNP** |  |  |  |  |  |
| 1. **Cardiology Consult** |  |  |  |  |  |
| 1. **Complete blood count** |  |  |  |  |  |
| 1. **Chest Radiograph** |  |  |  |  |  |
| 1. **EKG 12 LEAD** |  |  |  |  |  |
| 1. **Echocardiogram** |  |  |  |  |  |
| 1. **Troponin** |  |  |  |  |  |

Please indicate the reason(s) for your order (**check all that apply**):

|  | This reflects local practice patterns | Supervising physician would expect it | Data or guidelines supporting this decision | Decrease risk of lawsuits | To collect data for academic purposes | Rule out a potentially more dangerous condition | Would not order |
| --- | --- | --- | --- | --- | --- | --- | --- |
| 1. **Arterial blood gas** |  |  |  |  |  |  |  |
| 1. **Chem 7** |  |  |  |  |  |  |  |
| 1. **BNP** |  |  |  |  |  |  |  |
| 1. **Cardiology Consult** |  |  |  |  |  |  |  |
| 1. **Complete blood count** |  |  |  |  |  |  |  |
| 1. **Chest Radiograph** |  |  |  |  |  |  |  |
| 1. **EKG 12 LEAD** |  |  |  |  |  |  |  |
| 1. **Echocardiogram** |  |  |  |  |  |  |  |
| 1. **Troponin** |  |  |  |  |  |  |  |

**Clinical Vignette B, Part 1**

A 57 year old female, 50 pack-year former smoker with hypertension, COPD characterized as severe with recent PFT's showing FEV1/FVC of 0.63 and FEV1 value of 40% of the predicted value with three previous hospitalizations for COPD exacerbations presents after two days of shortness of breath associated with wheezing, increase in cough and sputum production as well as a sensation of chest tightness. Home medications include hydrochlorothiazide, tiotropium, salmeterol/fluticasone, and albuterol as needed. Vitals signs reveal a temperature of 37.4C, heart rate of 94, respiratory rate of 26, blood pressure 146/94, oxygen saturation of 84% on room air improving to 93% with nasal canula 4L per minute. Physical exam is significant for mild-moderate respiratory distress, use of accessory muscles of breathing without paradoxical chest wall motion, decreased air entry bilaterally in all lung fields. There are no crackles at the bases lower extremity edema or erythema. Her mental status remains intact with normal alertness and function.

Based on the findings above how often would you order the following:

|  | Always/ almost always | Most of the time | Some of the time | Rarely | Never |
| --- | --- | --- | --- | --- | --- |
| 1. **Arterial Blood Gas** |  |  |  |  |  |
| 1. **Basic metabolic panel (chem 7)** |  |  |  |  |  |
| 1. **BNP** |  |  |  |  |  |
| 1. **Chest Radiograph** |  |  |  |  |  |
| 1. **Complete blood count** |  |  |  |  |  |
| 1. **EKG 12 LEAD** |  |  |  |  |  |
| 1. **Troponin** |  |  |  |  |  |
| 1. **Urgent Cardiac Catheterization** |  |  |  |  |  |

Please indicate the reason(s) for your order (**check all that apply**):

|  | This reflects local practice patterns | Supervising physician would expect it | Data or guidelines supporting this decision | Decrease risk of lawsuits | To collect data for academic purposes | Rule out a potentially more dangerous condition | Would not order |
| --- | --- | --- | --- | --- | --- | --- | --- |
| 1. **Arterial Blood Gas** |  |  |  |  |  |  |  |
| 1. **Chem 7** |  |  |  |  |  |  |  |
| 1. **BNP** |  |  |  |  |  |  |  |
| 1. **Chest Radiograph** |  |  |  |  |  |  |  |
| 1. **Complete blood count** |  |  |  |  |  |  |  |
| 1. **EKG 12 LEAD** |  |  |  |  |  |  |  |
| 1. **Troponin** |  |  |  |  |  |  |  |
| 1. **Urgent Cardiac Catheterization** |  |  |  |  |  |  |  |

**Clinical Vignette C, Part 1**

68 year old female with a past medical history of hypertension, depression and non-small cell cancer of the lung diagnosed two years prior status post resection and chemotherapy with known metastatic disease to the liver and thoracic spine diagnosed 4 months prior is brought to the emergency room by her family for productive cough, fever, and lethargy. The patient was discharged from the hospital 6 weeks ago after admission for pathologic vertebral fracture. The patient’s physical function has steadily declined over the past 4 weeks, with increased difficulty ambulating, worsened pain and decreased oral intake. Over the past week, the patient has had cough, productive of green sputum, fevers to 102, shortness of breath and increasing malaise. The patient is taking morphine ER and morphine IR for pain, miralax, simvastatin and escitalopram. Vital signs reveal a temperature of 38.2, blood pressure of 108/66, heart rate of 112, respiratory rate of 24, oxygen saturation 94% on room air. Your exam reveals a cachectic, lethargic but arousable patient; rales are present in the right lower lung field; mild abdominal tenderness over right upper quadrant, no rebound; neurological exam is non-focal, AxO x 1, follows simple commands. The patient is full code without a formally identified health care proxy.

Based on the findings above how often would you order the following:

|  | Always/ almost always | Most of the time | Some of the time | Rarely | Never |
| --- | --- | --- | --- | --- | --- |
| 1. **Arterial Blood Gas** |  |  |  |  |  |
| 1. **Basic metabolic panel (chem 7)** |  |  |  |  |  |
| 1. **Blood cultures** |  |  |  |  |  |
| 1. **Chest Radiograph** |  |  |  |  |  |
| 1. **Complete blood count** |  |  |  |  |  |
| 1. **CT angiography of the Chest** |  |  |  |  |  |
| 1. **CT non-contrast of head** |  |  |  |  |  |
| 1. **EKG 12 LEAD** |  |  |  |  |  |
| 1. **Urine analysis and culture** |  |  |  |  |  |

Please indicate the reason(s) for your order (**check all that apply**):

|  | This reflects local practice patterns | Supervising physician would expect it | Data or guidelines supporting this decision | Decrease risk of lawsuits | To collect data for academic purposes | Rule out a potentially more dangerous condition | Would not order |
| --- | --- | --- | --- | --- | --- | --- | --- |
| 1. **Arterial Blood Gas** |  |  |  |  |  |  |  |
| 1. **Chem 7** |  |  |  |  |  |  |  |
| 1. **Blood cultures** |  |  |  |  |  |  |  |
| 1. **Chest Radiograph** |  |  |  |  |  |  |  |
| 1. **Complete blood count** |  |  |  |  |  |  |  |
| 1. **CT angiography of the Chest** |  |  |  |  |  |  |  |
| 1. **CT non-contrast of head** |  |  |  |  |  |  |  |
| 1. **EKG** |  |  |  |  |  |  |  |
| 1. **Urine analysis and culture** |  |  |  |  |  |  |  |

**Clinical Vignette D, Part 1**

A 72 year old male with a past medical history of hypertension, benign prostatic hyperplasia, and arthritis was brought to the emergency room after having two near-syncopal episodes at home one hour prior. The patient states that he was doing light work in his garage. When he rose from the ground, he immediately felt lightheaded, noted his vision went “black” and he fell to the floor without hitting his head or losing consciousness. He called his wife who helped him to his feet, but he again felt light-headed and slumped to the ground. At this time his wife states he was diaphoretic and confused. The wife called EMS. After several minutes the patient returned to his baseline mental status. Presently the patient reports feeling well. Prior and during the episodes he denied chest pain, palpitations, vertigo, headache, bowel or bladder incontinence, or shortness of breath. The patient takes carvedilol, hydrochlorothiazide, acetaminophen, and started alfuzosin one week prior. Vital signs reveal a temperature of 37.2, blood pressure of 124/68, HR 60 lying flat and 114/64, 64 standing, respiratory rate of 16, oxygen saturation 98% on RA. He is mildly overweight, in no apparent distress; moist mucous membranes; no carotid bruit; lungs are clear; heart is regular with no murmur; abdomen is soft, non-tender and non-distended; stool hemoccult negative, alert and oriented x3, CNII-XII intact and normal gait.

Based on the findings above how often would you order the following:

|  | Always/ almost always | Most of the time | Some of the time | Rarely | Never |
| --- | --- | --- | --- | --- | --- |
| 1. **Basic metabolic panel (chem 7)** |  |  |  |  |  |
| 1. **Cardiology consult** |  |  |  |  |  |
| 1. **CBC** |  |  |  |  |  |
| 1. **EKG 12 LEAD** |  |  |  |  |  |
| 1. **Further history** |  |  |  |  |  |
| 1. **Neurology consult** |  |  |  |  |  |
| 1. **Non contrast CT Head** |  |  |  |  |  |
| 1. **Troponin** |  |  |  |  |  |

Please indicate the reason(s) for your order (**check all that apply**):

|  | This reflects local practice patterns | Supervising physician would expect it | Data or guidelines supporting this decision | Decrease risk of lawsuits | To collect data for academic purposes | Rule out a potentially more dangerous condition | Would not order |
| --- | --- | --- | --- | --- | --- | --- | --- |
| 1. **Chem 7** |  |  |  |  |  |  |  |
| 1. **Cardiology consult** |  |  |  |  |  |  |  |
| 1. **CBC** |  |  |  |  |  |  |  |
| 1. **EKG 12 LEAD** |  |  |  |  |  |  |  |
| 1. **Further history** |  |  |  |  |  |  |  |
| 1. **Neurology consult** |  |  |  |  |  |  |  |
| 1. **Non contrast CT Head** |  |  |  |  |  |  |  |
| 1. **Troponin** |  |  |  |  |  |  |  |

**Clinical Vignette E, Part 1**

An 82-year-old female with a past medical history of hypertension, osteoporosis, and CKD stage 3, presents to the emergency room in July complaining of 3 days of fever, shortness of breath, and cough productive of yellow sputum. The patient also complains of malaise and decreased oral intake. She denies nausea, vomiting, diarrhea, chest pain, lower extremity swelling, syncope, recent travel, sick contacts or any recent hospitalization. She lives at home with her husband. She has never received a pneumococcal or influenza vaccine. She takes lisinopril, calcium, vitamin D, and alendronate. Physical exam shows a temperature of 38.6, blood pressure of 118/68, heart rate of 104, respiratory rate of 24, oxygen saturation 96% on room air. She is no apparent distress, mildly diaphoretic; dry mucous membranes, no oropharyngeal erythema; no cervical lymphadenopathy; absent breath sounds in the right lower lung field; tachycardic but regular with no murmur; abdomen is soft, non-tender and non-distended; no lower extremity edema, alert and oriented x3.

Based on the findings above how often would you order the following:

|  | Always/ almost always | Most of the time | Some of the time | Rarely | Never |
| --- | --- | --- | --- | --- | --- |
| 1. **Arterial blood gas** |  |  |  |  |  |
| 1. **Blood culture** |  |  |  |  |  |
| 1. **CBC with differential** |  |  |  |  |  |
| 1. **Chest x-ray** |  |  |  |  |  |
| 1. **d-dimer** |  |  |  |  |  |
| 1. **EKG 12 LEAD** |  |  |  |  |  |
| 1. **Troponin** |  |  |  |  |  |
| 1. **Urinalysis** |  |  |  |  |  |

Please indicate the reason(s) for your order (**check all that apply**):

|  | This reflects local practice patterns | Supervising physician would expect it | Data or guidelines supporting this decision | Decrease risk of lawsuits | To collect data for academic purposes | Rule out a potentially more dangerous condition | Would not order |
| --- | --- | --- | --- | --- | --- | --- | --- |
| 1. **Arterial blood gas** |  |  |  |  |  |  |  |
| 1. **Blood culture** |  |  |  |  |  |  |  |
| 1. **CBC with differential** |  |  |  |  |  |  |  |
| 1. **Chest x-ray** |  |  |  |  |  |  |  |
| 1. **d-dimer** |  |  |  |  |  |  |  |
| 1. **EKG 12 LEAD** |  |  |  |  |  |  |  |
| 1. **Troponin** |  |  |  |  |  |  |  |
| 1. **Urinalysis** |  |  |  |  |  |  |  |

Clinical Vignette F, Part 1

A 54-year-old male with a past medical history of hypertension and hyperlipidemia presents to the emergency room complaining of severe substernal chest pressure associated with shortness of breath, worsened by exertion and improved with rest. His symptoms started 45 minutes ago. He denies history of CAD. He takes his hypertension medication intermittently, but cannot remember the drug name. He is also taking simvastatin, but no aspirin. His pain continues to be excruciating at the time of his arrival to the hospital where he is given an aspirin 325mg tab. He denies history of smoking and family history is only significant for a mother who passed away from an MI at 71 years of age. On exam, the patient appears comfortable lying flat in bed, not in respiratory distress. Vital signs reveal a temperature of 37.2, with blood pressure is 147/94 in R arm and 145/92 in L arm, heart rate 88 and respiratory rate of 16 breaths per minute, oxygen saturation 99% on room air. His chest pain is not reproducible with palpation and his cardiac exam reveals a normal S1 and S2, no murmurs rubs or gallops. The remainder of the exam is unremarkable.

Based on the findings above how often would you order the following:

|  | Always/ almost always | Most of the time | Some of the time | Rarely | Never |
| --- | --- | --- | --- | --- | --- |
| 1. **Administer SL NTG to evaluate response** |  |  |  |  |  |
| 1. **Basic metabolic panel (chem 7)** |  |  |  |  |  |
| 1. **Chest x-ray** |  |  |  |  |  |
| 1. **CK-MB** |  |  |  |  |  |
| 1. **CT angiography of chest** |  |  |  |  |  |
| 1. **Echocardiogram** |  |  |  |  |  |
| 1. **EKG 12 lead** |  |  |  |  |  |
| 1. **PT/PTT** |  |  |  |  |  |
| 1. **Troponin** |  |  |  |  |  |

Please indicate the reason(s) for your order (**check all that apply**):

|  | This reflects local practice patterns | Supervising physician would expect it | Data or guidelines supporting this decision | Decrease risk of lawsuits | To collect data for academic purposes | Rule out a potentially more dangerous condition | Would not order |
| --- | --- | --- | --- | --- | --- | --- | --- |
| 1. **Administer SL NTG** |  |  |  |  |  |  |  |
| 1. **Chem 7** |  |  |  |  |  |  |  |
| 1. **Chest x-ray** |  |  |  |  |  |  |  |
| 1. **CK-MB** |  |  |  |  |  |  |  |
| 1. **CT angiography of chest** |  |  |  |  |  |  |  |
| 1. **Echocardiogram** |  |  |  |  |  |  |  |
| 1. **EKG 12 lead** |  |  |  |  |  |  |  |
| 1. **PT/PTT** |  |  |  |  |  |  |  |
| 1. **Troponin** |  |  |  |  |  |  |  |

Clinical Vignette G, Part 1

A 27 year old female with a past medical history of seasonal allergic rhinitis, eczema and asthma (4 lifetime hospitalizations for exacerbation, no history of intubation), displaced right ulnar fracture set in the ER 3 weeks ago was directly admitted to the hospital from clinic where she presented with severe shortness of breath. She reports that she has had a runny nose with congestion and a dry cough for the past 4 days with 2 days of wheezing and shortness of breath only mildly improved by nebulizer treatments at home. She reports no sick contacts at home and denies any new medications or exposures. She denies fever or chills. Review of systems reveals headache without neck stiffness or photophobia. Your physical exam shows a temperature of 37.6, blood pressure of 135/70, heart rate of 115, respiratory rate of 34. She is mildly agitated, unable to complete full sentences without pausing to breath, using accessory muscles, has mild oropharyngeal and nasal septal erythema without purulent discharge with chest exam significant for diffuse wheezing without crackles or transmitted bronchial sounds. Her exam is otherwise unremarkable.

Based on the findings above how often would you order the following:

|  | Always/ almost always | Most of the time | Some of the time | Rarely | Never |
| --- | --- | --- | --- | --- | --- |
| 1. **Arterial blood gas** |  |  |  |  |  |
| 1. **Chest x-ray** |  |  |  |  |  |
| 1. **Chest CT angiogram** |  |  |  |  |  |
| 1. **CBC** |  |  |  |  |  |
| 1. **D-dimer** |  |  |  |  |  |
| 1. **Peak expiratory flow** |  |  |  |  |  |
| 1. **Serial Pulse oximetry measurements** |  |  |  |  |  |

Please indicate the reason(s) for your order (**check all that apply**):

|  | This reflects local practice patterns | Supervising physician would expect it | Data or guidelines supporting this decision | Decrease risk of lawsuits | To collect data for academic purposes | Rule out a potentially more dangerous condition | Would not order |
| --- | --- | --- | --- | --- | --- | --- | --- |
| 1. **Arterial blood gas** |  |  |  |  |  |  |  |
| 1. **Chest x-ray** |  |  |  |  |  |  |  |
| 1. **Chest CT angiogram** |  |  |  |  |  |  |  |
| 1. **CBC** |  |  |  |  |  |  |  |
| 1. **D-dimer** |  |  |  |  |  |  |  |
| 1. **Peak expiratory flow** |  |  |  |  |  |  |  |
| 1. **Serial Pulse oximetry measurements** |  |  |  |  |  |  |  |

Clinical Vignette H, Part 1

A 77 year old female with a past medical history of hypothyroidism treated with synthroid and current smoker presents to the emergency room complaining of an abnormality in her vision. She reports that 2 days ago she had a similar episode lasting about a half hour during which she "couldn't see from the left eye." She chose not to seek medical attention since the problem resolved rapidly. She now reports recurrence of her symptoms with complete loss of vision in the left that lasted for almost an hour and has only completely resolved within the last ten minutes. She denies headache, focal weakness or dysarthria. She denies any fevers and her review of systems is otherwise negative. A physical exam reveals a temperature of 36.7, blood pressure 117/74, heart rate of 78, respiratory rate of 14, unremarkable cardiovascular exam including regular rate and rhythm, no carotid bruit and no murmurs, negative pulmonary and abdominal exams with 2+ symmetrical strength in her extremities, normal sensation, negative Babinski sign bilaterally, PERRLA with EOMI and 20/20 vision in each eye tested separately. Her retinal exam is unremarkable.

Based on the findings above how often would you order the following:

|  | Always/ almost always | Most of the time | Some of the time | Rarely | Never |
| --- | --- | --- | --- | --- | --- |
| 1. **CBC, basic metabolic panel** |  |  |  |  |  |
| 1. **Brain imaging CT or MRI** |  |  |  |  |  |
| 1. **Determination of institutional Carotid Endarterctomy Complication Rate** |  |  |  |  |  |
| 1. **Doppler ultrasound of the neck** |  |  |  |  |  |
| 1. **EKG 12 lead** |  |  |  |  |  |
| 1. **Fasting lipid panel** |  |  |  |  |  |
| 1. **Troponin** |  |  |  |  |  |
| 1. **Transthoracic Echocardiogram (TTE)** |  |  |  |  |  |

Please indicate the reason(s) for your order (**check all that apply**):

|  | This reflects local practice patterns | Supervising physician would expect it | Data or guidelines supporting this decision | Decrease risk of lawsuits | To collect data for academic purposes | Rule out a potentially more dangerous condition | Would not order |
| --- | --- | --- | --- | --- | --- | --- | --- |
| 1. **CBC, basic metabolic panel** |  |  |  |  |  |  |  |
| 1. **Brain imaging CT or MRI** |  |  |  |  |  |  |  |
| 1. **Determination of Carotid Endarterctomy Complication Rate** |  |  |  |  |  |  |  |
| 1. **Doppler U/S of the neck** |  |  |  |  |  |  |  |
| 1. **EKG** |  |  |  |  |  |  |  |
| 1. **fasting lipid panel** |  |  |  |  |  |  |  |
| 1. **Troponin** |  |  |  |  |  |  |  |
| 1. **TTE** |  |  |  |  |  |  |  |

**----------STOP----------**

**You have now completed section I of the survey. This booklet will now be collected and you will receive a second booklet which contains the second portion of each clinical vignette.**

**Section II: Part 2 of Clinical Vignettes A-H**

In this section, part 2 of the vignettes will reveal the results of the indicated tests based on the patient presentation in part 1. You will now be asked to indicate your likelihood of arranging for a series of further tests and treatments, again ordered alphabetically. **Please check all that apply.** Again you will be asked to indicate the reason for your decisions. As a reminder, part 1 of the vignette is also included. At the end of the clinical vignettes you will be asked to answer several demographic questions.

Clinical Vignette A, Part 1

A 75 year old male with a past medical history of NYH Class III congestive heart (Echo from admission 6 months prior shows RV and LV dilation and an LVEF of 28%) on optimal medical management, status post AICD placement, chronic kidney disease stage III, coronary artery disease status post a non-drug eluting stent to the mid-LAD 2 years prior, and hypertension is admitted through the emergency room with 7 days of shortness of breath, decreased exercise tolerance and increased lower extremity edema. The patient denies chest pain. Vital signs reveal a temperature of 37.2, blood pressure of 135/84, heart rate of 92, respiratory rate of 20, oxygen saturation 93% on room air. Physical exam is significant for mild respiratory distress, bilateral crackles at lung bases, +JVD, and warm extremities with 1+ pitting lower extremity edema bilaterally. This patient has had 3 admissions for congestive heart failure in the past 6 months.

Clinical Vignette A, Part 2

Diagnostic evaluation reveals:

BNP is 2500 (normal <100). WBC count is 9.5 (normal 4.5-11.0), PMNs 76% (normal 40-78%). Hemoglobin is 11.4 (normal 11.7-15.0) Creatinine is 2.4 (normal 0.5-1.3), baseline 1.7. K^+^ is 4.2 (normal 3.5-5.0). Troponin 0.0 (normal <0.5). EKG shows LVH, Q waves in lateral leads, unchanged from prior. Chest x-ray shows cardiomegaly and bibasilar haziness, infiltrate cannot be excluded.

Based on these findings, how often would you order the following treatments and/or additional diagnostic evaluations:

|  | Always/ almost always | Most of the time | Some of the time | Rarely | Never |
| --- | --- | --- | --- | --- | --- |
| 1. **Antibiotics for community acquired pneumonia (CAP)** |  |  |  |  |  |
| 1. **Consult to cardiology** |  |  |  |  |  |
| 1. **Consult to nephrology** |  |  |  |  |  |
| 1. **Daily monitoring of urine input/output, weight and electrolytes** |  |  |  |  |  |
| 1. **Goals of care discussion** |  |  |  |  |  |
| 1. **Intravenous loop diuretics** |  |  |  |  |  |
| 1. **Parenteral ionotropes** |  |  |  |  |  |
| 1. **Serial cardiac enzymes** |  |  |  |  |  |
| 1. **Supplemental oxygen** |  |  |  |  |  |
| 1. **Urine electrolytes** |  |  |  |  |  |

For each of the above please indicate the reason(s) for your order (**check all that apply**):

|  | This reflects local practice patterns | Supervising physician would expect it | Data or guidelines supporting this decision | Decrease risk of lawsuits | To collect data for academic purposes | Rule out a potentially more dangerous condition | Would not order |
| --- | --- | --- | --- | --- | --- | --- | --- |
| 1. **Antibiotics for CAP** |  |  |  |  |  |  |  |
| 1. **Consult to cardiology** |  |  |  |  |  |  |  |
| 1. **Consult to nephrology** |  |  |  |  |  |  |  |
| 1. **Daily monitoring I/Os, weight and electrolytes** |  |  |  |  |  |  |  |
| 1. **Goals of care discussion** |  |  |  |  |  |  |  |
| 1. **Intravenous loop diuretics** |  |  |  |  |  |  |  |
| 1. **Parenteral ionotropes** |  |  |  |  |  |  |  |
| 1. **Serial cardiac enzymes** |  |  |  |  |  |  |  |
| 1. **Supplemental oxygen** |  |  |  |  |  |  |  |
| 1. **Urine electrolytes** |  |  |  |  |  |  |  |

**Clinical Vignette B, Part 1**

A 57 year old female, 50 pack-year former smoker with hypertension, COPD characterized as severe with recent PFT's showing FEV1/FVC of 0.63 and FEV1 value of 40% of the predicted value with three previous hospitalizations for COPD exacerbations presents after two days of shortness of breath associated with wheezing, increase in cough and sputum production as well as a sensation of chest tightness. Home medications include hydrochlorothiazide, tiotropium, salmeterol/fluticasone, and albuterol as needed. Vitals signs reveal a temperature of 37.4C, heart rate of 94, respiratory rate of 26, blood pressure 146/94, oxygen saturation of 84% on room air improving to 93% with nasal canula 4L per minute. Physical exam is significant for mild-moderate respiratory distress, use of accessory muscles of breathing without paradoxical chest wall motion, decreased air entry bilaterally in all lung fields. There are no crackles at the bases, lower extremity edema or erythema. Her mental status remains intact with normal alertness and function.

**Clinical Vignette B, Part 2**

Diagnostic evaluation reveals:

The radiograph shows hyperinflation of both fields without localized infiltrate, and no cardiomegaly. EKG shows normal sinus rhythm with LVH pattern without right heart strain pattern or ST-T changes. Arterial blood gas shows pH 7.30 (normal 7.35-7.43) with pCO2 65 (normal 35-45) and pO2 of 60 (normal 80-105). WBC count is 10.5 (normal 4.5-11), hemoglobin of 16 (normal 11.7-15.0) and HCO2 of 30 (normal 22-30).

Based on these findings how often would you order the following treatments and/or additional diagnostic evaluations:

|  | Always/ almost always | Most of the time | Some of the time | Rarely | Never |
| --- | --- | --- | --- | --- | --- |
| 1. **Antibiotics** |  |  |  |  |  |
| 1. **Chest CT with angiography** |  |  |  |  |  |
| 1. **Daily peak flow measurement** |  |  |  |  |  |
| 1. **Goals of care discussion** |  |  |  |  |  |
| 1. **Inhaled beta-2 agonist** |  |  |  |  |  |
| 1. **Inhaled Ipratropium** |  |  |  |  |  |
| 1. **Non-invasive mechanical ventilation** |  |  |  |  |  |
| 1. **Pulmonology Consult** |  |  |  |  |  |
| 1. **Sputum culture with sensitivity** |  |  |  |  |  |
| 1. **Steroids Oral** |  |  |  |  |  |
| 1. **Steroids IV** |  |  |  |  |  |

For each of the above please indicate the reason(s) for your order (**check all that apply**):

|  | This reflects local practice patterns | Supervising physician would expect it | Data or guidelines supporting this decision | Decrease risk of lawsuits | To collect data for academic purposes | Rule out a potentially more dangerous condition | Would not order |
| --- | --- | --- | --- | --- | --- | --- | --- |
| 1. **Antibiotics** |  |  |  |  |  |  |  |
| 1. **Chest CT with angiography** |  |  |  |  |  |  |  |
| 1. **Daily peak flow measure** |  |  |  |  |  |  |  |
| 1. **Goals of care discussion** |  |  |  |  |  |  |  |
| 1. **Inhaled beta-2 agonist** |  |  |  |  |  |  |  |
| 1. **Inhaled Ipratropium** |  |  |  |  |  |  |  |
| 1. **Non-invasive mechanical ventilation** |  |  |  |  |  |  |  |
| 1. **Pulmonology Consult** |  |  |  |  |  |  |  |
| 1. **Sputum culture with sensitivity** |  |  |  |  |  |  |  |
| 1. **Steroids Oral** |  |  |  |  |  |  |  |
| 1. **Steroids IV** |  |  |  |  |  |  |  |

**Clinical Vignette C, Part 1**

68 year old female with a past medical history of hypertension, depression and non-small cell cancer of the lung diagnosed two years prior status post resection and chemotherapy with known metastatic disease to the liver and thoracic spine diagnosed 4 months prior is brought to the emergency room by her family for productive cough, fever, and lethargy. The patient was discharged from the hospital 6 weeks ago after admission for pathologic vertebral fracture. The patient’s physical function has steadily declined over the past 4 weeks, with increased difficulty ambulating, worsened pain and decreased oral intake. Over the past week, the patient has had cough, productive of green sputum, fevers to 102, shortness of breath and increasing malaise. The patient is taking morphine ER and morphine IR for pain, miralax, simvastatin and escitalopram. Vital signs reveal a temperature of 38.2, blood pressure of 108/66, heart rate of 112, respiratory rate of 24, oxygen saturation 94% on room air. Your exam reveals a cachectic, lethargic but arousable patient; rales are present in the right lower lung field; mild abdominal tenderness over right upper quadrant, no rebound; neurological exam is non-focal, AxO x 1, follows simple commands. The patient is full code without a formally identified health care proxy.

**Clinical Vignette C, Part 2**

Diagnostic evaluation reveals:

Labs significant for HGB 10.4 (normal 11.7-15.0), WBC of 17.8 (normal 4.5-11.0) with PMNs of 88% (normal 40-78%), platelets of 413 (normal 150-450), sodium 131 (normal 135-145), potassium 4.5 (normal 3.5-5.0), chloride 98 (normal 96-108), bicarbonate 17 (normal 22-32), BUN 52 (normal 10-30), creatinine 2.1 (normal 0.5-1.3) (baseline 0.7), glucose 121 (normal 66-199). BCx and UCx are in lab. UA shows +ketones, no blood, 1+ WBC, no bacteria, +leukocyte esterase, no nitrites. CXR PA and LL shows L perihilar mass and an infiltrate in the right lower lobe. EKG shows sinus tachycardia with no ischemic changes.

Based on these findings how often would you order the following treatments and/or additional diagnostic evaluations

|  | Always/ almost always | Most of the time | Some of the time | Rarely | Never |
| --- | --- | --- | --- | --- | --- |
| 1. **Antibiotics for community acquired pneumonia (CAP)** |  |  |  |  |  |
| 1. **Antibiotics for healthcare associated pneumonia (HCAP)** |  |  |  |  |  |
| 1. **CT non-contrast of the head** |  |  |  |  |  |
| 1. **Fluid resuscitation** |  |  |  |  |  |
| 1. **Goals of care discussion** |  |  |  |  |  |
| 1. **MICU consult** |  |  |  |  |  |
| 1. **Palliative care consult** |  |  |  |  |  |
| 1. **Respiratory fluid culture** |  |  |  |  |  |
| 1. **Supplemental oxygen** |  |  |  |  |  |
| 1. **Urine electrolytes** |  |  |  |  |  |

For each of the above please indicate the reason(s) for your order (**check all that apply**):

|  | This reflects local practice patterns | Supervising physician would expect it | Data or guidelines supporting this decision | Decrease risk of lawsuits | To collect data for academic purposes | Rule out a potentially more dangerous condition | Would not order |
| --- | --- | --- | --- | --- | --- | --- | --- |
| 1. **Antibiotics for CAP** |  |  |  |  |  |  |  |
| 1. **Antibiotics for HCAP** |  |  |  |  |  |  |  |
| 1. **CT non-contrast of the head** |  |  |  |  |  |  |  |
| 1. **Fluid resuscitation** |  |  |  |  |  |  |  |
| 1. **Goals of care discussion** |  |  |  |  |  |  |  |
| 1. **MICU consult** |  |  |  |  |  |  |  |
| 1. **Palliative care consult** |  |  |  |  |  |  |  |
| 1. **Respiratory fluid culture** |  |  |  |  |  |  |  |
| 1. **Supplemental oxygen** |  |  |  |  |  |  |  |
| 1. **Urine electrolytes** |  |  |  |  |  |  |  |

**Clinical Vignette D, Part 1**

A 72 year old male with a past medical history of hypertension, benign prostatic hyperplasia, and arthritis was brought to the emergency room after having two near-syncopal episodes at home one hour prior. The patient states that he was doing light work in his garage. When he rose from the ground, he immediately felt lightheaded, noted his vision went “black” and he fell to the floor without hitting his head or losing consciousness. He called to his wife who helped him to his feet, but he again felt light-headed and slumped to the ground. At this time his wife states he was diaphoretic and confused. The wife called EMS. After several minutes the patient returned to his baseline mental status. Presently the patient reports feeling well. Prior and during the episodes he denied chest pain, palpitations, vertigo, headache, bowel or bladder incontinence, or shortness of breath. The patient takes carvedilol, hydrochlorothiazide, acetaminophen, and started alfuzosin one week prior. Vital signs reveal a temperature of 37.2, blood pressure of 124/68, HR 60 lying flat and 114/64, 64 standing, respiratory rate of 16, oxygen saturation 98% on RA. He is mildly overweight, in no apparent distress; moist mucous membranes; no carotid bruit; lungs are clear; heart is regular with no murmur; abdomen is soft, non-tender and non-distended; stool hemoccult negative, alert and oriented x3, CNII-XII intact and normal gait.

**Clinical Vignette D, Part 2**

Diagnostic evaluation reveals:

Further questioning reveals that the patient has no family history of sudden cardiac death. This is the first near-syncopal episode he has experienced. His usual exercise tolerance is 7-8 blocks and he is only limited by knee pain. Labs are significant for HGB 12.6 (normal 11.7-15.0), WBC of 8.4 (normal 4.5-11) with PMNs of 68% (normal 40-78%), platelets of 358 (normal 150-450), sodium 141 (normal 135-145), potassium 4.5 (normal 3.5-5.0), chloride 108 (normal 96-108), bicarbonate 23 (normal 22-32), BUN 13 (normal 10-30), creatinine 0.9 (normal 0.5-1.3), glucose 121 (normal 66-199). EKG shows normal sinus rhythm at a rate of 55 beats per minute, first degree block is noted with a PR interval of 0.24, no ST elevations, depressions or Q waves, normal R wave progression.

Based on these findings how often would you order the following treatments and/or additional diagnostic evaluations:

|  | Always/ almost always | Most of the time | Some of the time | Rarely | Never |
| --- | --- | --- | --- | --- | --- |
| 1. **Continuous telemetry monitoring (III)** |  |  |  |  |  |
| 1. **Decrease dose of alfuzosin (I)** |  |  |  |  |  |
| 1. **Echocardiogram (NR)** |  |  |  |  |  |
| 1. **Exercise Stress test (III)** |  |  |  |  |  |
| 1. **Outpatient Holter monitoring (NR)** |  |  |  |  |  |
| 1. **Switch to different alpha-blocker (I)** |  |  |  |  |  |
| 1. **Tilt table test (III)** |  |  |  |  |  |
| 1. **Troponin level x 3 (NR)** |  |  |  |  |  |

For each of the above please indicate the reason(s) for your order (**check all that apply**):

|  | This reflects local practice patterns | Supervising physician would expect it | Data or guidelines supporting this decision | Decrease risk of lawsuits | To collect data for academic purposes | Rule out a potentially more dangerous condition | Would not order |
| --- | --- | --- | --- | --- | --- | --- | --- |
| 1. **Continuous telemetry monitoring** |  |  |  |  |  |  |  |
| 1. **Decrease dose of alfuzosin** |  |  |  |  |  |  |  |
| 1. **Echocardiogram** |  |  |  |  |  |  |  |
| 1. **Exercise Stress test** |  |  |  |  |  |  |  |
| 1. **Outpatient Holter monitoring** |  |  |  |  |  |  |  |
| 1. **Switch to different alpha-blocker** |  |  |  |  |  |  |  |
| 1. **Tilt table test** |  |  |  |  |  |  |  |
| 1. **Troponin level x 3** |  |  |  |  |  |  |  |

**Clinical Vignette E, Part 1**

An 82-year-old female with a past medical history of hypertension, osteoporosis, and CKD stage 3, presents to the emergency room in July complaining of 3 days of fever, shortness of breath, and cough productive of yellow sputum. The patient also complains of malaise and decreased oral intake. She denies nausea, vomiting, diarrhea, chest pain, lower extremity swelling, syncope, recent travel, sick contacts or any recent hospitalization. She lives at home with her husband. She has never received a pneumococcal or influenza vaccine. She takes lisinopril, calcium, vitamin D, and alendronate. Physical exam shows a temperature of 38.6, blood pressure of 118/68, heart rate of 104, respiratory rate of 24, oxygen saturation 96% on room air. She is no apparent distress, mildly diaphoretic; dry mucous membranes, no oropharyngeal erythema; no cervical lymphadenopathy; absent breath sounds in the right lower lung field; tachycardic but regular with no murmur; abdomen is soft, non-tender and non-distended; no lower extremity edema, alert and oriented x3.

**Clinical Vignette E, Part 2**

Diagnostic evaluation reveals:

Labs are significant for HGB 15.6 (normal 11.7-15.0), WBC of 16.7 (normal 4.5-11.0) with PMNs of 91% (normal 40-78%), platelets of 558 (normal 150-450), sodium 146 (normal 135-145), potassium 4.5 (normal 3.5-5.0), chloride 112 (normal 96-108), bicarbonate 23 (normal 22-32), BUN 28 (normal 10-30), creatinine 1.7 (normal 0.5-1.3) (baseline 1.2) , glucose 111 (normal 66-199). Chest x-ray shows likely right middle and lower lobe infiltrate without effusion. Blood cultures were sent to the lab.

Based on these findings how often would you order the following treatments and/or additional diagnostic evaluations:

|  | Always/ almost always | Most of the time | Some of the time | Rarely | Never |
| --- | --- | --- | --- | --- | --- |
| 1. **Antibiotics for community acquired PNA (CAP)** |  |  |  |  |  |
| 1. **CT –chest high resolution non-contrast** |  |  |  |  |  |
| 1. **Foley catheter** |  |  |  |  |  |
| 1. **Influenza Vaccine to be given at d/c** |  |  |  |  |  |
| 1. **Isotonic intravenous fluids** |  |  |  |  |  |
| 1. **MICU consult** |  |  |  |  |  |
| 1. **Pneumococcal vaccine to be given at D/C** |  |  |  |  |  |
| 1. **Renal consult** |  |  |  |  |  |
| 1. **Sputum culture** |  |  |  |  |  |
| 1. **Urine legionella antigen** |  |  |  |  |  |

For each of the above please indicate the reason(s) for your order (**check all that apply**):

|  | This reflects local practice patterns | Supervising physician would expect it | Data or guidelines supporting this decision | Decrease risk of lawsuits | To collect data for academic purposes | Rule out a potentially more dangerous condition | Would not order |
| --- | --- | --- | --- | --- | --- | --- | --- |
| 1. **Antibiotics for CAP** |  |  |  |  |  |  |  |
| 1. **CT –chest high resolution non-contrast** |  |  |  |  |  |  |  |
| 1. **Foley catheter** |  |  |  |  |  |  |  |
| 1. **Influenza Vaccine at d/c** |  |  |  |  |  |  |  |
| 1. **Isotonic intravenous fluids** |  |  |  |  |  |  |  |
| 1. **MICU consult** |  |  |  |  |  |  |  |
| 1. **Pneumococcal vaccine at D/C** |  |  |  |  |  |  |  |
| 1. **Renal consult** |  |  |  |  |  |  |  |
| 1. **Sputum culture** |  |  |  |  |  |  |  |
| 1. **Urine legionella antigen** |  |  |  |  |  |  |  |

Clinical Vignette F, Part 1

A 54-year-old male with a past medical history of hypertension and hyperlipidemia presents to the emergency room complaining of severe substernal chest pressure associated with shortness of breath, worsened by exertion and improved with rest. His symptoms started 45 minutes ago. He denies history of CAD. He takes his hypertension medication intermittently, but cannot remember the drug name. He is also taking simvastatin, but no aspirin. His pain continues to be excruciating at the time of his arrival to the hospital where he is given an aspirin 325mg tab. He denies history of smoking and family history is only significant for a mother who passed away from an MI at 71 years of age. On exam, the patient appears comfortable lying flat in bed, not in respiratory distress. Vital signs reveal a temperature of 37.2, with blood pressure is 147/94 in R arm and 145/92 in L arm, heart rate 88 and respiratory rate of 16 breaths per minute, oxygen saturation 99% on room air. His chest pain is not reproducible with palpation and his cardiac exam reveals a normal S1 and S2, no murmurs rubs or gallops. The remainder of the exam is unremarkable.

Clinical Vignette F, Part 2

Diagnostic evaluation reveals:

His initial EKG shows no ST segment abnormalities but T wave inversions in V4-V6 that are new when compared to a previous EKG. His initial troponin I is 2.4 (normal <0.5). His chest pain has improved but has not resolved after administration of nitroglycerin sublingually. Repeat EKG shows deepening T-waves in the same pattern. Troponin peaks at 4.4 with no further EKG changes.

Based on these findings how often would you order the following treatments and/or additional diagnostic evaluations

|  | Always/ almost always | Most of the time | Some of the time | Rarely | Never |
| --- | --- | --- | --- | --- | --- |
| 1. **ACE inhibitor within 24 hours** |  |  |  |  |  |
| 1. **Bed rest with continuous EKG monitoring** |  |  |  |  |  |
| 1. **Clopidogrel 300mg stat** |  |  |  |  |  |
| 1. **Continuous oxygen therapy** |  |  |  |  |  |
| 1. **CCU consult** |  |  |  |  |  |
| 1. **Diagnostic percutaneous transluminal coronary angiography (ptca)** |  |  |  |  |  |
| 1. **Echocardiography** |  |  |  |  |  |
| 1. **Exercise stress test** |  |  |  |  |  |
| 1. **Oral beta blocker within 24 hours** |  |  |  |  |  |
| 1. **SL NTG for ongoing chest pain** |  |  |  |  |  |
| 1. **Unfractionated or Low Molecular Weight Heparin** |  |  |  |  |  |

For each of the above please indicate the reason(s) for your order (**check all that apply**):

|  | This reflects local practice patterns | Supervising physician would expect it | Data or guidelines supporting this decision | Decrease risk of lawsuits | To collect data for academic purposes | Rule out a potentially more dangerous condition | Would not order |
| --- | --- | --- | --- | --- | --- | --- | --- |
| 1. **ACE inhibitor within 24 hours** |  |  |  |  |  |  |  |
| 1. **Bed rest with continuous EKG monitoring** |  |  |  |  |  |  |  |
| 1. **Clopidogrel 300mg stat** |  |  |  |  |  |  |  |
| 1. **Continuous oxygen therapy** |  |  |  |  |  |  |  |
| 1. **CCU consult** |  |  |  |  |  |  |  |
| 1. **Diagnostic PTCA** |  |  |  |  |  |  |  |
| 1. **Echocardiography** |  |  |  |  |  |  |  |
| 1. **Exercise stress test** |  |  |  |  |  |  |  |
| 1. **Oral β-blocker within 24 hrs** |  |  |  |  |  |  |  |
| 1. **SL NTG for ongoing chest pain** |  |  |  |  |  |  |  |
| 1. **Unfractionated or Low Molecular Weight Heparin** |  |  |  |  |  |  |  |

Clinical Vignette G, Part 1

A 27 year old female with a past medical history of seasonal allergic rhinitis, eczema and asthma (4 lifetime hospitalizations for exacerbation, no history of intubation), displaced right ulnar fracture set in the ER 3 weeks ago was directly admitted to the hospital from clinic where she presented with severe shortness of breath. She reports that she has had a runny nose with congestion and a dry cough for the past 4 days with 2 days of wheezing and shortness of breath only mildly improved by nebulizer treatments at home. She reports no sick contacts at home and denies any new medications or exposures. She denies fever or chills. Review of systems reveals headache without neck stiffness or photophobia. Your physical exam shows a temperature of 37.6, blood pressure of 135/70, heart rate of 115, respiratory rate of 34. She is mildly agitated, unable to complete full sentences without pausing to breath, using accessory muscles, has mild oropharyngeal and nasal septal erythema without purulent discharge with chest exam significant for diffuse wheezing without crackles or transmitted bronchial sounds. Her exam is otherwise unremarkable.

Clinical Vignette G, Part 2

Diagnostic evaluation reveals:

Peak expiratory flow is 35% of the value predicted, oxygen saturation on pulse oximetry is 89%, blood gas reveals pH 7.45 (normal 7.35-7.43) with pCO2 35 (normal 35-45) and pO2 of 60 (normal 80-105).

Based on these findings how often would you order the following treatments and/or additional diagnostic evaluations:

|  | Always/ almost always | Most of the time | Some of the time | Rarely | Never |
| --- | --- | --- | --- | --- | --- |
| 1. **Administration of antibiotics** |  |  |  |  |  |
| 1. **Inhaled ipratropium bromide** |  |  |  |  |  |
| 1. **Intravenous magnesium** |  |  |  |  |  |
| 1. **Intravenous systemic corticosteroids** |  |  |  |  |  |
| 1. **Oral systemic corticosteroids** |  |  |  |  |  |
| 1. **Pulmonology consult** |  |  |  |  |  |
| 1. **Repeat peak expiratory flow** |  |  |  |  |  |
| 1. **Short acting beta agonist therapy** |  |  |  |  |  |
| 1. **Supplemental oxygen** |  |  |  |  |  |

Please indicate the reason(s) for your order (**check all that apply**):

|  | This reflects local practice patterns | Supervising physician would expect it | Data or guidelines supporting this decision | Decrease risk of lawsuits | To collect data for academic purposes | Rule out a potentially more dangerous condition | Would not order |
| --- | --- | --- | --- | --- | --- | --- | --- |
| 1. **Administration of antibiotics** |  |  |  |  |  |  |  |
| 1. **Inhaled ipratropium bromide** |  |  |  |  |  |  |  |
| 1. **Intravenous magnesium** |  |  |  |  |  |  |  |
| 1. **Intravenous systemic corticosteroids** |  |  |  |  |  |  |  |
| 1. **Oral systemic corticosteroids** |  |  |  |  |  |  |  |
| 1. **Pulmonology consult** |  |  |  |  |  |  |  |
| 1. **Repeat peak expiratory flow** |  |  |  |  |  |  |  |
| 1. **Short acting beta agonist therapy** |  |  |  |  |  |  |  |
| 1. **Supplemental oxygen** |  |  |  |  |  |  |  |

Clinical Vignette H, Part 1

A 77 year old female with a past medical history of hypothyroidism treated with synthroid and current smoker presents to the emergency room complaining of an abnormality in her vision. She reports that 2 days ago she had a similar episode lasting about a half hour during which she "couldn't see from the left eye." She chose not to seek medical attention since the problem resolved rapidly. She now reports recurrence of her symptoms with complete loss of vision in the left that lasted for almost an hour and has only completely resolved within the last ten minutes. She denies headache, focal weakness or dysarthria. She denies any fevers and her review of systems is otherwise negative. A physical exam reveals a temperature of 36.7, blood pressure 117/74, heart rate of 78, respiratory rate of 14, unremarkable cardiovascular including regular rate and rhythm, no carotid bruit and no murmurs, negative pulmonary and abdominal exams with 2+ symmetrical strength in her extremities, normal sensation, negative Babinski sign bilaterally, PERRLA with EOMI and 20/20 vision in each eye tested separately. Her retinal exam is unremarkable.

Clinical Vignette H, Part 2

Diagnostic evaluation reveals:

CBC and BMP are unremarkable. EKG reveals normal sinus rhythm with no signs of ischemia. Head CT without contrast and MRI show no evidence of ischemia. Doppler ultrasound of the neck shows left carotid artery stenosis of 60% with 30% stenosis on the right. Your institution’s carotid endarterctomy perioperative complication rate is 4%. Fasting lipids are remarkable for an LDL of 190 (normal <130).

Based on these findings how often would you order the following treatments and/or additional diagnostic evaluations

|  | Always/ almost always | Most of the time | Some of the time | Rarely | Never |
| --- | --- | --- | --- | --- | --- |
| 1. **Anti hypertensive at the time of discharge** |  |  |  |  |  |
| 1. **Anti platelet therapy** |  |  |  |  |  |
| 1. **Carotid endarterectomy** |  |  |  |  |  |
| 1. **Hospitalization** |  |  |  |  |  |
| 1. **Oral warfarin** |  |  |  |  |  |
| 1. **Smoking cessation counseling** |  |  |  |  |  |
| 1. **Statin at the time of discharge** |  |  |  |  |  |

Please indicate the reason(s) for your order (**check all that apply**):

|  | This reflects local practice patterns | Supervising physician would expect it | Data or guidelines supporting this decision | Decrease risk of lawsuits | To collect data for academic purposes | Rule out a potentially more dangerous condition | Would not order |
| --- | --- | --- | --- | --- | --- | --- | --- |
| 1. **Anti hypertensive at the time of discharge** |  |  |  |  |  |  |  |
| 1. **Anti platelet therapy** |  |  |  |  |  |  |  |
| 1. **Carotid endarterectomy** |  |  |  |  |  |  |  |
| 1. **Hospitalization** |  |  |  |  |  |  |  |
| 1. **Oral warfarin** |  |  |  |  |  |  |  |
| 1. **Smoking cessation counseling** |  |  |  |  |  |  |  |
| 1. **Statin at the time of discharge** |  |  |  |  |  |  |  |

**----------STOP----------**

**You have now completed section II of the survey. Please continue to the next page to complete section III.**

**Section III: Baseline Survey Measures**

**Please provide us with a few demographic details as well as your opinions regarding your practice style.**

**Baseline Demographics**

1. Gender 🞎 Male 🞎 Female
2. Age 🞎 <40 🞎 40-60 🞎>60

Graduate Medical Education

1. Name of the city where you did complete or will complete your internal medicine residency______________
2. Please indicate the type of program in which you trained or are training
   - Categorical Internal Medicine
   - Internal Medicine/Pediatrics
   - Internal Medicine/Emergency Medicine
   - Internal Medicine/Neurology
   - Primary Care Internal Medicine
   - Internal Medicine Research Track
   - Other_________________________
3. Please describe the nature of the primary hospital where you will complete or did complete your residency

- Private
- Public
- Veterans Administration

**Current and future certifications**

1. Are you board certified in an Internal Medicine subspecialty?

- yes
- no

1. If you are not certified in an Internal Medicine subspecialty, are you currently in or do you plan to enroll in an Internal Medicine subspecialty fellowship?

- yes
- no

**Current or future employment**

1. Please indicate your current or, if still in training, the intended geographic setting in which you plan to practice.

- Metropolitan area
- Suburb
- Rural area

1. Please describe your current or, if still in training, intended primary clinical responsibilities.
   - Outpatient primary care
   - Outpatient specialty care
   - Inpatient general internal medicine service
   - Inpatient consult
   - Inpatient specialty care service
   - Inpatient critical care
   - Other____________________

**Please indicate your level of agreement with the following statements.**

|  | **Strongly Agree** | **Agree** | **Neutral** | **Disagree** | **Strongly Disagree** |
| --- | --- | --- | --- | --- | --- |
| **My Clinical Practice Patterns are Substantially Influenced By:** |  |  |  |  |  |
| 1. Accumulated research and evidence. |  |  |  |  |  |
| 1. Gaining experience with new tests and treatments. |  |  |  |  |  |
| 1. Previous residency training hospital culture |  |  |  |  |  |
| 1. Local hospital culture. |  |  |  |  |  |
| 1. Patient demands. |  |  |  |  |  |
| 1. Medical malpractice concerns. |  |  |  |  |  |
| 1. Individual concerns to ensure profitable practice. |  |  |  |  |  |
| 1. Hospital concerns to ensure profitable practice. |  |  |  |  |  |
| **The Following Utilize Care at High Rates:** |  |  |  |  |  |
| 1. My individual clinical practice. |  |  |  |  |  |
| 1. My colleagues’ clinical practice |  |  |  |  |  |
| 1. My hospital’s clinical practice. |  |  |  |  |  |
| **Comparative Effectiveness Research Should:** |  |  |  |  |  |
| 1. Evaluate treatment strategies |  |  |  |  |  |
| 1. Evaluate diagnostic test strategies |  |  |  |  |  |
| **To Deserve Widespread Use, Diagnostic Tests and Treatments Should:** |  |  |  |  |  |
| 1. Cost less than $50,000 |  |  |  |  |  |
| 1. Cost less than $100,000 |  |  |  |  |  |
| 1. Cost less than $500,000 |  |  |  |  |  |
| per Quality Adjusted Life Year (QALY) saved. |  |  |  |  |  |

1. Please rank the sources of information (1 being the most important, 7 being the least important) which you use to inform your clinical practice:

__ Attending local CME activities

__ Attending national or international CME activities

__ Colleagues’ experiences

__ Other Internet and print medical resources

__ Personal experience

__ Reading peer-reviewed journal articles

__ Reading practice guidelines or consensus statements

**Thank you for your time.**
